# Comparison of surgical outcome in impingement syndrome with and without stiff shoulder

**DOI:** 10.4103/0019-5413.40255

**Published:** 2008

**Authors:** Jin-Young Park, Dilbans Singh Pandher, Gi-Hyuk Moon, Moon-Jib Yoo, Sung Tae Lee

**Affiliations:** Department of Orthopedic Surgery, Konkuk University School of Medicine, Seoul, Korea; 1 Department of Orthopedic Surgery, Pohang St. Mary's Hospital, Korea; 2 Department of Orthopedic Surgery, Dankook University College of Medicine, Korea

**Keywords:** Acromioplasty, arthroscopy, impingement syndrome, manipulation, stiff shoulder

## Abstract

**Background:**

In impingment syndrome with associated stiff shoulder the general protocol of management is to conservatively treat the stiff shoulder followed by operative treatment of the impingement syndrome. This consecutive prospective study was carried out to evaluate the functional outcome of surgical management for impingement syndrome associated with stiff shoulder and to compare the results with surgical management of impingement syndrome alone.

**Materials and Methods:**

We evaluated a total of 100 patients with impingement syndrome, consisting of 76 patients with impingement syndrome alone (Group A) and 24 patients of stiff shoulder associated with impingement syndrome (Group B). Group A patients were treated by subacromial decompression alone and Group B patients were treated by closed manipulation under anesthesia followed by subacromial decompression.

**Results:**

According to the American Shoulder and Elbow Surgeons (ASES) evaluation score satisfactory results were obtained in 80% patients of Group A and 67% patients of Group B, while for patients with diabetes [(*n* = 18), Group A (n = 11), Group B (n = 7)] satisfactory results were achieved in 82% of patients of Group A(9/11) and 43% of Group B(3/7). Overall, Group B patients had a lower range of motion for external rotation postoperatively, thus indicating that procedures to improve the external rotation, such as a release of the rotator interval or anterior capsule, might be considered in conjunction with other surgical procedures in patients with impingement syndrome with associated stiffness to further improve functional outcome.

**Conclusion:**

Acromioplasty can be performed in stiff shoulder associated with impingement syndrome without fears of further worsening of stiffness from adhesions with the exposed raw undersurface of acromian. Patients with diabetes mellitus and shoulder stiffness tend to have poor clinical outcomes and must receive appropriate counseling preoperatively.

## INTRODUCTION

The hallmark of stiff shoulder is restriction of passive glenohumoral joint mobility often resulting in loss of active range of movements because of associated pain. This, at times makes it difficult to differentiate it from decreased range of motion because of pain in impingement syndrome. Impingement syndrome often restricts the shoulder motion and interferes with patient's ability to perform daily activities. Neer^1^ recommended non-surgical treatment initially for shoulder impingement syndrome, including for those with limited range of motion. Non-surgical treatment, however, may not be effective if the shoulder joint remains stiff for an extended period and passive range of motion is restricted.^2^

Surgical intervention is a valid alternative in such clinical presentations, although there are some concerns associated with the procedure, for example acromioplasty is reported to result in raw acromial undersurface that can lead to postoperative adhesions.^3^ There are no reports detailing results of manipulation under anesthesia followed by subacromial decompression without the use of operative capsular release in impingement syndrome with stiffness. In this retrospective study we compared the effectiveness of closed manipulation and arthroscopic subacromial decompression in management of impingement syndrome with associated shoulder stiffness, with that of subacromial decompression alone in patients with impingement syndrome without associated shoulder stiffness.

## MATERIALS AND METHODS

We evaluated 105 consecutive patients over the duration of one year with impingement syndrome who failed to respond to conservative management. Five patients were not available for follow-up and hence were excluded, leaving 100 patients who were followed up for a minimum period of two years. These included 76 patients with impingement syndrome alone (Group A) and 24 patients with impingement syndrome associated with stiff shoulder (Group B).

**Figure 1:**
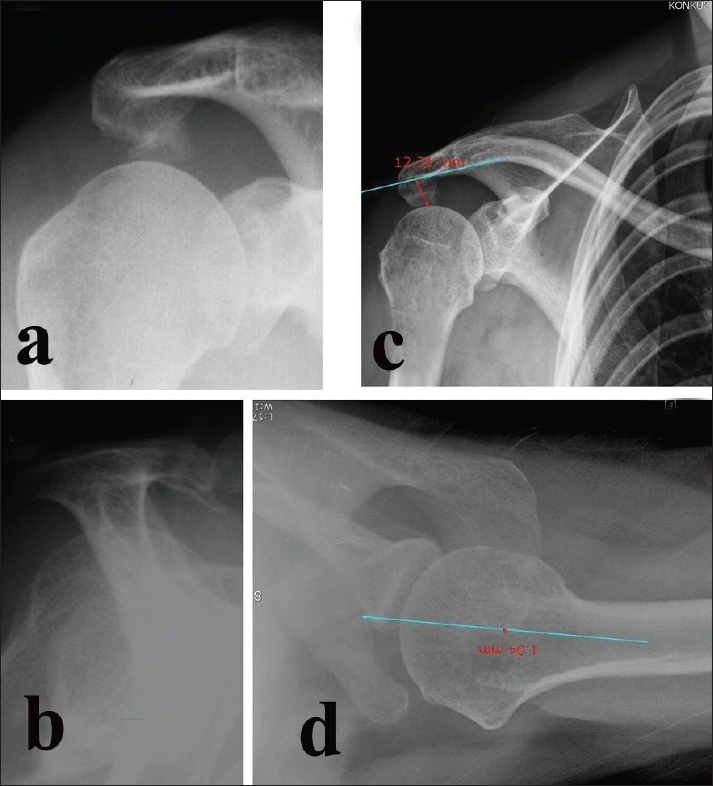
Showing (a) acromial spur in 30° caudal tilt view X-ray; (b) downward acromial spur in supraspinatus outlet view; (c) and (d) showing measurement of amount of acromial resection required in X-rays

**Figure 2:**
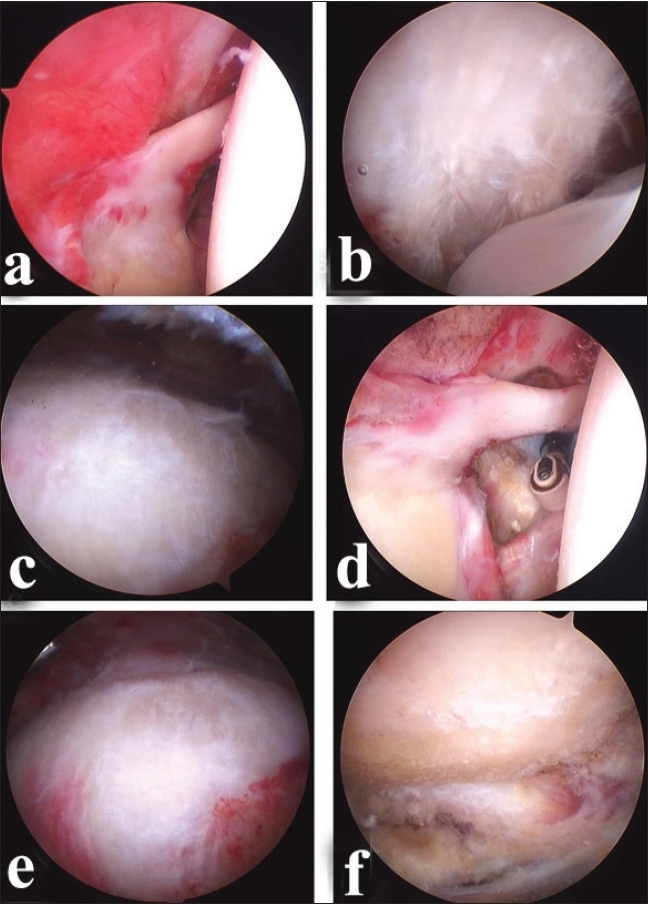
Showing arthroscopic pictures of (a) synovitis in the glenohumeral joint (b) subacromial fraying along the coracoacromial arch caused by impingement syndrome (c) fraying on the rotator cuff caused by impingement syndrome (d) synovectomy and rotator interval resection performed using radiofrequency device (e) arthroscopic subacromial decompression (f) bursal side rotator cuff after debridement to the fraying rotator Cuff

We noted the shoulder range of motion(ROM), tender points vis-´-vis at the insertion of supraspinatus, acromioclavicular joint, bicipital groove, coracoid process and anteroinferior glenoid margin and impingement sign (Neer, Hawkins). Physical examination also included Lhermitte test and Spurling test). Finally, impingement injection test was performed. Radiological investigations included X-rays for shoulder and cervical spine, Magentic Resonance Imaging (MRI) or ultrasonography for the shoulder joint as indicated. As per the preoperative history, physical and radiological examination, we excluded patients with other specific pathologies like glenohumeral arthritis, calcific tendinitis, full or partial thickness tear of rotator cuff, rupture of biceps tendon, cervical arthritis, herniated intervertebral disc of cervical spine, trauma around the shoulder and brain lesions manifesting as pain or decreased range of motion at shoulder joint. Indications for surgery included shoulder pain or disability refractory to supervised non-surgical treatment including non-steroidal anti-inflammatory medications, local steroid injections and an intensive physical therapy exercise program for a minimum duration of six months [Table 1].

**Table 1:** Demographic findings of patients in the impingement syndrome with and without stiffness groups

	ISWOS*	ISWS†
No. of patients	76 cases	24 cases
Average age	51 years (31-76 years)	54 years (40-70 years)
Male:Female	29:47	8:16
Symptom duration	27 months (6-150 months)	30 months (5-240 months)
Improvement after impingement test	67% (50-100%)	65% (50-100%)
Rate of diabetes mellitus	13%	29%
Fasting blood glucose of diabetic patients	223 mg/dl (116-375 mg/dl)	206 mg/dl (120-409 mg/dl)

* ISWOS: impingement syndrome without stiffness;

† ISWS: impingement syndrome with stiffness

At the initial visit the diagnosis was made and the patient underwent clinical examination for evaluation of range of movements, the American Shoulder and Elbow Surgeons (ASES) functional assessment score and pain assessment on visual analog scale (VAS) to obtain objective measure of their shoulder disability once the diagnosis of impingement syndrome was established.

Subacromial impingement injection test was performed for the diagnosis of impingement syndrome, by injecting 6 to 8 mL of 2% lidocaine into the subacromial bursa. Visual analog scale (VAS) was used to assess pain at the subacromial space before and 15 min after the injection. The ASES scale was used to measure pain and function.^4^ The result was considered positive when the pain score decreased by more than 50% in the VAS [Table 1].^5^ Impingement syndrome with stiff shoulder was defined as forward elevation less than 100° and external rotation at the side less than 30°^2^. After the impingement injection test, patients in whom range of motion improved more than the level defined within the impingement syndrome with stiffness were categorized under Group A. This is because some patients show pseudo-limitation of motion of the shoulder due to pain during shoulder movements. After impingement injection test, some patients show improvement in the shoulder range of motion due to pain relief offered by the anesthetic drug.

### Intervention:

In patients with stiffness a closed manipulation under anesthesia was performed prior to arthroscopic examination. An assistant reaching under the patient's back stabilized the axillary border of the scapula. The operator grasped the humerus to be manipulated high in the axilla to avoid excessive leverage. After obtaining sufficient forward elevation, the humerus was gradually abducted to 90° and then gently externally rotated. Manipulation was done in the state of external rotation at the side. Finally, it was internally rotated with minimal force. We repeated this procedure until a satisfactory range of motion was achieved.^6^ During closed manipulation, a definite and sharp popping or snapping of the capsular constriction and sudden increase in the range of motion were observed in 20 cases, with the remaining four undergoing gradual increase in range of motion of shoulder due to slow plastic deformation of the capsule.

Arthroscopic surgery was performed in the beach-chair position with balanced suspension.^7^ The amount of acromioplasty was determined in the suprascapular outlet view and anteroposterior scapular view with 30° of caudal tilt. After arthroscopic acromioplasty, arthroscopic distal clavicle resection for acromioclavicular tenderness and arthritis [in patients who complained about acromioclavicular (AC) joint pain and who had tenderness on the AC joint] was performed in eight patients with shoulder stiffness and in six without stiffness.^7^

Within 24 h of surgery, passive forward elevation of the shoulder using a pulley and string was encouraged. Physical therapy regimen including active range-of-motion exercises, as tolerated, was started from the first postoperative day. Patients were evaluated preoperatively, postoperatively at monthly intervals for six months and at three-monthly intervals thereafter till the final follow-up. The mean follow-up period was 32 months in Group A and 33 months in Group B.

### Statistical analysis:

The student t-test was used to analyze the significances of ages and range of movement and the Mann-Whitney U test was used for analysis of pain and ASES evaluation score. Evaluations scores of pre- and post-surgery assessment were compared to assess improvement after surgical intervention and *p* values of less than 0.05 were considered statistically significant.

## RESULTS

Group A (impingement syndrome without stiffness) comprised 76 patients with a mean age of 51 years and Group B (impingement syndrome with stiffness) comprised 24 patients with a mean age of 54 years, thus providing 24% incidence of stiff shoulder in association with impingement syndrome refractory to conservative treatment in our series. Of these, 11 (14.5%) patients in Group A and seven (29%) patients in Group B had diabetes mellitus, with a mean fasting blood glucose level of 223 mg/dL and 206 mg/dL respectively [Table 1]. This difference in the incidence of diabetes in the stiff shoulder group and impingement syndrome alone group is significant (*P* < 0.05). The average duration of symptoms was 28 months (range, 5-240 months). Prior to treatment, the mean pain relief according to VAS after the impingement injection test was 67% in patients without stiff shoulder and 65% in patients with stiff shoulder [Table 1]. Postoperative pain and the ASES functional score improved significantly in both the groups compared to baseline values (*P* < 0.05). Preoperatively Group B patients had more severe pain and worse ASES functional score as compared to Group A. Postoperative pain score and ASES scores were not significantly different (*P* > 0.05) between the two groups [Table 2].

**Table 2:** Comparison of ASES functional scores and range of motion in impingement syndrome with and without stiffness groups

	Preoperative	Postoperative
ISWOS*		
Pain	7 (5-10)‡	1 (0-5)‡
ASES score	33 (5-60)‡	91 (53-100)‡
Forward flexion	127°(60°-160°)‡	154°(140°-170°)
External rotation at the side	53° (10°-90°)‡	71° (30°-80°)‡
External rotation at 90° abduction	68° (20°-90°)‡	79° (60°-90°)‡
Internal rotation	T_12_ (T_7_-buttock)‡	T_8_ (T_5_-L_4_)‡
ISWS†		
Pain	8 (5-10)‡	1 (0-5)
ASES score	24 (3-60)‡	88 (57-100)
Forward flexion	89° (60°-100°)‡	151° (135°-170°)
External rotation at the side	12° (-10°-30°)‡	63° (30°-80°)‡
External rotation at 90° abduction	34° (0°-60°)‡	78° (70°-80°)
Internal rotation	L_3_ (L_1_-buttock)‡	T_9_ (T_6_-L_1_)

* ISWOS = impingement syndrome without stiffness;

† ISWS = impingement syndrome with stiffness;

‡ *P* < 0.05 between ISWOS and ISWS

We used the patient ASES scores to determine satisfactory outcomes [Table 2]. Those patients who achieved excellent (91 to 100 points) or good (81 to 90 points) results were considered to have a satisfactory outcome. Thus, 16 (67%) of the patients with stiffness (Group B) and 61 (80%) of the patients without stiffness (Group A) had satisfactory outcomes according to ASES evaluation score. A satisfactory outcome in diabetic patients was achieved in 3 (43%) of those with stiffness and 9 (82%) of those without stiffness [Table 4]. Patient satisfaction was 83% in Group B and 93% in the Group A [Table 3]. Forward elevation, external rotation at the side and internal rotation improved in both groups postoperatively. Group B (with stiffness) had a lower range of motion for external rotation as compared to Group A postoperatively (*P* > 0.05). No other statistically significant differences in range of motion were observed between the groups postoperatively [Table 2].

**Table 4:** Results for patients with diabetes mellitus in the impingement syndrome with and without stiffness groups

	ISWOS*		ISWS†	

	Preoperative	Postoperative	preoperative	Postoperative
ASES score	28 (12-58)	88 (65-100)	29 (3-60)	80 (57-100)
Excellent		8 cases (72.7%)		3 cases (42.85%)
Good		1 case (9.1%)		0 case (-)
Fair		2 cases (18.2%)		2 cases (28.57%)
Poor		0 case (-)		2 cases (28.57%)
Patient satisfaction		81%		57%

* ISWOS: impingement syndrome without stiffness;

† ISWS = impingement syndrome with stiffness; Excellent → 91 to 100 points; Good → 81 to 90 points; Fair → 71 to 80 points; Poor → 70 points or less

**Table 3:** Patient outcome and satisfaction in the impingement syndrome with and without stiffness groups

ASES score	Number of patients with ISWOS*	Number of patients with ISWS†
Excellent (91 to 10 points)	53 cases (69.7%)	14 cases (58.3%)
Good (81 to 90 points)	8 cases (10.5%)	2 cases (8.3%)
Fair (71 to 80 points)	10 cases (13.2%)	6 cases (25%)
Poor (70 points or less)	5 cases (6.6%)	2 cases (8.3%)

* ISWOS = impingement syndrome without stiffness;

† ISWS = impingement syndrome with stiffness

## DISCUSSION

Differentiation between impingement syndrome alone, stiff shoulder alone and impingement syndrome with shoulder stiffness can sometimes be a diagnostic challenge for a shoulder surgeon since pain and limitation of motion are common clinical presentations of these three entities. The prevalence of frozen shoulder or adhesive capsulitis in the general population is estimated to be approximately 2%, but is more frequently observed in patients with diabetes, cervical disease, hyperthyroidism and intrathoracic disease. Neviaser^8^ reported that adhesive capsulitis is a disease of the capsule and synovial membrane around the humeral head and that Stage 1 adhesive capsulitis mimics shoulder impingement syndrome.^9^ Several authors have reported that stiffness of the shoulder joint may develop in impingement syndrome.^1^,^10^–^15^ When stiffness occurs simultaneously with impingement syndrome, it is difficult to differentiate this condition from primary frozen shoulder. Neer^1^ reported that frozen shoulder pain unrelieved by subacromial analgesic injection is caused by stiffness of the glenohumeral joint. Clinically, stiffness associated with impingement syndrome is characterized by limited forward elevation, internal rotation and adduction across the body.^11^

Various treatment methods are described in the literature for the management of stiff shoulder which include local steroid injection and physiotherapy,^16^–^19^ manipulation under anesthesia,^10^,^12^,^14^,^15^,^17^,^20^ joint distention^21^ or arthroscopic capsular release.^17^,^22^–^25^ Hydraulic distension^12^ in an attempt to rupture the joint capsule can be carried out under local anesthesia; however, it is often poorly tolerated because of pain. Arthroscopic distension^12^ of the anterior structures has been reported, though it has limitations because of the restricted volume of the glenohumoral joint, which makes it difficult to enter the correct space without causing damage to the articular surface. Open division is well tolerated and avoids some of the above mentioned complications but does make it more difficult to carry out early physiotherapy because of restrictions while the wound heals. We performed closed manipulation of the joint under anesthesia which allows closed rupture of the capsular contractures while not interfering with the early active mobilization. With continuous local analgesic delivery into the joint postoperatively, it is a very well tolerated procedure.

However, manipulation under anesthesia has been associated with complications such as fracture, tendon rupture and brachial plexus injury^26^–^28^ for which cautious and skillful execution of the technique is important. We did not come across any such complication.

Several authors have reported that stiff shoulder in diabetics had a poorer outcome.^6^,^16^,^18^,^24^,^29^ Our study also showed that patients with shoulder stiffness and diabetes mellitus had a relatively poor outcome and unsatisfactory results. These patients who cannot be treated successfully with conservative treatment should be considered for operative treatment.

Patients who suffered from impingement pain with stiff shoulder rarely needed more aggressive treatment such as the global capsular release reported by Goldberg.^23^ Although we did not use the arthroscopic capsular release technique in patients with stiffness, postoperative forward elevation and internal rotation improved to the similar motion range in both groups. The average postoperative external rotation of Group B (with impingement syndrome and stiff shoulder), however, was worse than that of the Group A (without stiffness). We therefore agree with Bennett's^22^ report that patients with impingement syndrome and stiff shoulder should be treated with a procedure to improve the external rotation, such as a release of the rotator interval or anterior capsular release in conjunction with the subacromial decompression.

Within 24 h of surgery, passive forward elevation of the shoulder using a pulley was encouraged. Physical therapy regimen including active range-of-motion exercises, as tolerated, was started from the first postoperative day. We believe this helped in prevention of postoperative subacromial adhesions, which are a feared cause of recurrence of stiffness after acromioplasty in patients with preoperative stiffness.

Five patients could not be followed up in our series, since one patient died because of myocardial infarction and another died of lung cancer and three patients did not turn up for postoperative checkups since they migrated to other cities. The association of diabetes was significantly higher in the group with stiffness as compared to the impingement syndrome alone group (*P* < 0.05). This might be the reason for poor results in patients with diabetes since they are predisposed to stiffness due to the disease itself.

We believe that it is important to treat both the pathologies i.e. impingement syndrome and stiff shoulder simultaneously, since staged management prolongs the suffering of the patient due to a long interval between the two procedures. Dodenhoff *et al.*,^30^ reported failure of manipulation under anesthesia for pain relief in stiff shoulder with associated impingement syndrome which required subsequent treatment for the impingement syndrome.

## CONCLUSION

Conservative treatment may not be effective in addressing the impingement syndrome in patients with associated shoulder stiffness. For these patients the technique of manipulation followed by arthroscopic subacromial decompression offers good pain relief and satisfactory functional recovery, although patients with stiff shoulder may not regain the full range of motion as compared to patients of impingement syndrome without stiff shoulder.

Additional studies are required to define which procedures result in the best clinical outcomes. Procedures to improve the external rotation, such as a release of the rotator interval or anterior capsular release, should be performed in conjunction with other surgical procedures in patients with impingement syndrome and stiffness.
